# Challenges and opportunities for the future of monoclonal antibody development: Improving safety assessment and reducing animal use

**DOI:** 10.1080/19420862.2017.1324376

**Published:** 2017-05-05

**Authors:** Fiona Sewell, Kathryn Chapman, Jessica Couch, Maggie Dempster, Shawn Heidel, Lise Loberg, Curtis Maier, Timothy K. Maclachlan, Marque Todd, Jan Willem van der Laan

**Affiliations:** a UK National Centre for the Replacement, Refinement & Reduction of Animals in Research (NC3Rs), London, UK; b Genentech, Inc., South San Francisco, CA, USA; c GlaxoSmithKline, King of Prussia, PA, USA; d Covance Laboratories Ltd, Greenfield, IN, USA; e AbbVie, Department R46G, North Chicago, IL, USA; f Novartis, Cambridge, MA, USA; g Pfizer, Science Center Drive, La Jolla, CA, USA; h Division of Toxicology, Leiden Academic Center for Drug Research, Leiden University, Leiden, The Netherlands; i Medicines Evaluation Board, Utrecht, The Netherlands

**Keywords:** Attrition, in vitro technologies, monoclonal antibodies (mAbs), 3Rs, safety assessment

## Abstract

The market for biotherapeutic monoclonal antibodies (mAbs) is large and is growing rapidly. However, attrition poses a significant challenge for the development of mAbs, and for biopharmaceuticals in general, with large associated costs in resource and animal use. Termination of candidate mAbs may occur due to poor translation from preclinical models to human safety. It is critical that the industry addresses this problem to maintain productivity. Though attrition poses a significant challenge for pharmaceuticals in general, there are specific challenges related to the development of antibody-based products. Due to species specificity, non-human primates (NHP) are frequently the only pharmacologically relevant species for nonclinical safety and toxicology testing for the majority of antibody-based products, and therefore, as more mAbs are developed, increased NHP use is anticipated. The integration of new and emerging *in vitro* and *in silico* technologies, e.g., cell- and tissue-based approaches, systems pharmacology and modeling, have the potential to improve the human safety prediction and the therapeutic mAb development process, while reducing and refining animal use simultaneously. In 2014, to engage in open discussion about the challenges and opportunities for the future of mAb development, a workshop was held with over 60 regulators and experts in drug development, mechanistic toxicology and emerging technologies to discuss this issue. The workshop used industry case-studies to discuss the value of the *in vivo* studies and identify opportunities for *in vitro* technologies in human safety assessment. From these and continuing discussions it is clear that there are opportunities to improve safety assessment in mAb development using non-animal technologies, potentially reducing future attrition, and there is a shared desire to reduce animal use through minimised study design and reduced numbers of studies.

## Introduction

**Figure 1.**
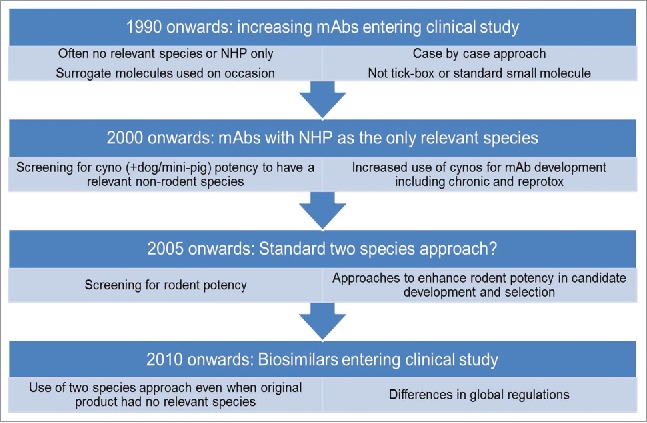
Use of animals in mAb development and change in practice over time.

The market for protein-based biotherapeutics is large and is still growing rapidly. In 2015, 27% (12/45) of the drugs approved by the Food and Drug Administration (FDA) were biologic products, the highest number yet.^1^ The largest group of biologics are antibody-based, mainly mAbs. There are 61 antibody-based products currently approved and in review in Europe and the US (as of October 2016).^2^^,^^3^ Recently, biosimilar products (a biologic medicinal product that contains a highly similar but not identical version of the original active substance of an already authorized original biologic medicinal product) have also entered the market. The European Medicines Agency (EMA) has approved 21 biosimilars to date,^2^ including the first mAb biosimilar of infliximab, Inflectra (infliximab-dyyb), which received regulatory approval in Europe in September 2013.^4^ In March 2015, the FDA approved its first biosimilar product (Zarxio™, filgrastim-sndz, Sandoz), which was followed by the first US mAb biosimilar approval, in April 2016 (for Inflectra).^5^^,^^6^ It is well documented that animal use in the development of mAbs, as well as other protein-based biotherapeutics and biosimilars, poses unique challenges to those associated with new chemical entities, and that these challenges have evolved over time and have contributed to attrition (Fig. 1).^7-11^ While attrition owing to nonclinical safety events occurs less frequently for mAbs than for small molecules, these events certainly happen and underscore the importance of a thorough safety evaluation in relevant biologics systems.^12^ Due to the species specificity of many products, non-human primates (NHPs) have been used for nonclinical safety and toxicology testing for the majority of antibody-based products as they are often the only species in which the mAb binds and has the desired pharmacological effect. However, there are often fundamental differences between primate and human physiology, and consequently there are often still deficiencies in the translation of NHP study results to human. For example, this may occur if the target does not play a role (or is redundant) in normal physiology in NHPs, or in cases where the target is still present, but has a different role or downstream effect in primate compared with human. In such situations, studies in NHP may therefore be of limited value to human risk assessment. Alternatively, mouse target knockout phenotypes can be used for hazard identification in place of the NHP, or surrogate molecules can be used in rodent species to demonstrate safety and efficacy.

Toxicological science is also advancing rapidly across the public and private sectors. For example, in the chemicals industry adverse outcome pathways (AOPs), data from the USA's ToxCast program and exposure-based modeling are being applied to human risk assessment, shifting the focus toward human-specific mechanisms of action and pathways-based approaches.^13^ There is regulatory interest in these approaches nationally and internationally at the Organization for Economic Co-operation and Development level (OECD).^14^ Many of these approaches, in combination with technologies emerging from the research base, also show the potential to transform the pharmaceutical industry. For example, the ambitious collaboration between the National Institutes of Health's (NIH) National Center for Advancing Translational Science, Defense Advanced Research Projects Agency (DARPA) and FDA in the US has committed $150 million (from NIH and DARPA) over 5 y to develop tissue chips that mimic human physiology to screen for safe, effective drugs (http://www.ncats.nih.gov/tissuechip).^15^ The FDA also recently announced a multi-year research and development agreement with Emulate Inc., a company founded by researchers at the Wyss Institute, to evaluate their “Organs-on-Chips” technology in laboratories at the agency's Center for Food Safety and Applied Nutrition.^16^ The availability of new technologies alongside recent publications that have questioned whether the use of NHPs adds scientific value to the development of mAbs suggests that the timing is right to review the current biotherapeutic mAb development paradigm.^17^^,^^18^

In 2014, to engage in open discussion about the challenges and opportunities for the future of mAb development, a workshop was held with experts in drug development, mechanistic toxicology and emerging technologies such as cell and tissue-based approaches, systems pharmacology and modeling. The aims were to: 1) identify the knowledge and data gaps if scientists were to rely more heavily on the emerging technologies for the development of biotherapeutics; 2) determine how to optimise prediction of human safety by better understanding of mechanisms/target pharmacology; and 3) gain more value from fewer *in vivo* studies. The 60 participants included current FDA and European Union (EU) regulators and representatives from the pharmaceutical, biotechnology and contract research industries. A selection of current state-of-the-art techniques were showcased and discussed with a view to how these could be applied either now or in the future to improve the safety assessment of mAbs. Although, it was recognized it may be some time before any of these can be used successfully for decision-making in drug development, the workshop provided a unique opportunity to mine the vast knowledge and experience of this group to gain a consensus perspective on a future vision for safety assessment in mAb development. This paper provides an overview of the discussions that began at the workshop, descriptions of real-life industry case studies with consideration of the value of the *in vivo* and *in vitro* studies, and a plan for future work developed by the authors based on the output of the workshop and recent developments in the field.

## The workshop

### Emerging technologies

The most recent estimated figure for the cost of getting a drug to market is almost $2.6 billion^19^ and between 2008 and 2010 productivity of the pharmaceutical industry was at an all-time low despite the introduction of the first wave of biotechnology derived products in the 1990s. There is some recent evidence that R&D productivity has turned a corner and the industry is sustainable again.^20^^,^^21^ However, attrition, which may be due to lack of efficacy as well as lack of translational safety, is still a huge problem, costing an estimated $1.4 billion per drug.^19^ For mAbs, the lack of cross-reactivity in rodents may contribute to attrition as there are limited opportunities to study drug candidates in rodent pharmacology models. It is critical for the industry to reduce attrition in order for the increase in productivity to continue.

Many technologies are currently being used to reduce attrition in candidate screening for both efficacy and safety, including stem cells, cardiac assays, and *in silico* models. However, many of these are being used for small molecules, often to assess off-target toxicity, rather than for the screening of biotherapeutics.^22^ One reason might be that such technologies are not relevant for mAb development because off-target toxicity rarely occurs and the toxicity is primarily related to specific on-target pharmacology. If this is true, the question remains as to whether these or similar technologies could be modified or developed to address questions that are more suited to biotherapeutics and, if so, how? Perhaps such technologies will need to be more case-specific, dependent on the binding target of the product.

Emerging technologies with possible relevance to biotherapeutic development were selected for discussion at the workshop and included: 1) ‘organs-on-chips’ types of *in vitro* technologies; 2) systems pharmacology and *in silico* modeling; 3) *in vitro* human immune models, as showcased by VaxDesigns's MIMIC® technology;^23^ and 4) human pluripotent stem cells as a tool for developmental biology, as showcased by Stemina Biomarker Discovery's devTOX™ discovery assay.^24^

The name ‘organ-on-a-chip’ refers to microfluidic cell culture devices that contain continuously perfused chambers inhabited by living cells arranged to simulate tissue- and organ-level physiology. The promise that ‘organs-on-chips’ offer to drug development has been well documented, but yet to be fully realized or adopted.^25^^,^^26^ This may be because these models are unable to fully represent the *in vivo* situation, to recreate a fully functioning organ outside of a living body, due to the more complex interplay between systems and processes at the whole animal level. Current research around the world is focused on the creation of reproducible systems that are representative of human disease states and also remain functional over a relevant time period to support target qualification and proof-of-concept studies. One of the most attractive components of the technology is the potential to generate genetically diverse ‘chips’ that may be used in clinical trial settings. The technology also enables high-resolution, real-time imaging and *in vitro* analysis of biochemical, genetic and metabolic activities of living human cells in a functional tissue and organ context.

Systems biology approaches aim to integrate the quantitative relationships between RNA, protein levels and metabolites to offer new insights into the function and behavior of organs, tissues and cells. Systems pharmacology describes an approach that links systems biology, pharmacology, medicinal chemistry and bioinformatics, and enables the development of models that predict and explain how drugs interact with biologic components. Modeling approaches are highly specific for the system(s) they describe and the questions being asked, thus, although they have shown value in defining potential on-target toxicities of new molecular entities, there is currently limited experience with using such approaches in the development of biologics.^27-29^ Increased application of systems pharmacology and modeling for biologics could improve the characterization of the target (including target expression levels and expected pharmacokinetic/pharmacodynamic effects in *in vivo* studies), provide better data integration, and support the potential to reduce animal use. In part, the development of relevant *in vitro* assays with quantitative readouts in human primary cells and organs-on-chips will be instrumental in refining/applying these models for biologics. One successful example of an *in vitro* technique that has been applied to better characterize mAbs is the *in vitro* Comparative Immunogenicity Assessment (IVCIA).^30^ This was developed as a tool for predicting potential relative immunogenicity of biotherapeutic mAbs as a screening and prioritization tool, to differentiate mAbs and detect differential immunogenicity as a result of aggregation, which has been shown to potentially enhance cytokine secretion and T-cell proliferation response in healthy volunteers.^31^

## Application of emerging technologies to real-life industry case studies

The assertion by van Meer et al. that NHPs do not necessarily add scientific value to the mAb development process is due mainly to the assumption that most mAb toxicity is related to exaggerated pharmacology and that such pharmacologically-mediated adverse effects could therefore be predicted from *in vitro* studies alone.^17^^,^^18^ However, since the time of the van Meer et al. publication, more exceptions to this assumption have been reported, in part as a result from data-sharing initiatives and workshops. One of the criticisms of publications reviewing current practice in how mAbs are developed is that they are often based solely on drugs that have been through regulatory review. Often, this approach is taken because regulatory dossiers (e.g., European public assessment reports, EU and FDA pharmacology and toxicology reviews) offer the only publically available information to assess. However, this leads to bias because conclusions are based on a limited sub-set of drugs, without representation of drugs that are terminated during development due to identified safety and toxicity issues. The drugs that are accepted for first-in-human (FIH) clinical studies are believed to be relatively safe drugs, as safety concerns such as severe toxicity would have been assessed non-clinically. To address this gap in available information, unpublished and published industry case-studies were gathered and analyzed to determine whether emerging technologies could have been used to predict nonclinical or clinical outcomes.

Several case-studies were selected for discussion at the workshop to enable a variety of targets and challenges to be debated in breakout groups. The same questions were asked for each case study and are listed in Table 1. Not all questions were relevant for all case studies and only relevant questions were answered in each breakout group.

**Table 1.** Questions for case studies addressed during the breakout sessions.

1. Were the effects observed in the preclinical studies conducted to support the first-in-human (FIH) study predictable based on the mechanism of action (MOA)?
a) Which effects were predictable based on the MOA? Please describe.
b) Which effects were not predictable? Please describe.
c) Could the non-predictable effects be attributed to either a) a background lesion in the animal used or b) a consequence of immunogenicity in the animal?
d) Do you think the use of a wider range of *in vitro* approaches may have aided the prediction of the effects observed (both those based on MOA and other non-MOA effects)?
i. If your response is yes, please describe in further detail.
ii. If your response is no, please describe the knowledge and data gaps, or other issues, which make such an *in vitro* approach problematic.
2. Prediction of clinical dose/exposure: Could human serum levels/PK be predicted using *in vitro* data, *in vivo* data from rodents, or a combination of these?
If your response is no:
i. Please describe the knowledge and data gaps, or other issues, which make such an approach problematic.
ii. What are your reasons for requiring data from NHPs to predict the clinical dose/exposure?
3. Could this product have safely entered into clinical trials on the basis of *in vitro* approaches driven by the MOA?
4. What additional effects were observed in longer-term general toxicity studies that were not observed in the studies conducted to support the FIH study?
a) Which of the additional effects were predictable based on the MOA? Please describe.
b) Which effects were not predictable? Please describe.
c) Did the additional effects impact on clinical decision making during clinical development e.g., design of clinical studies (inclusion, exclusion criteria, clinical doses, clinical monitoring)?
5. Could this product have progressed through clinical development and to registration on the basis of *in vitro* approaches?
a) If your response is no, could the use of *in vitro* approaches lead to a reduced need for *in vivo* studies? Please summarize your discussions.
6. If studies to assess toxicity to reproduction were conducted for the product, were the observed effects predictable based on the MOA?
a) Which effects were predictable based on the MOA?
b) Which effects were not predictable?
c) Could *in vitro* approaches have provided sufficient information for clinical risk communication and management?
7. In your view are juvenile toxicity studies warranted for this product to support pediatric indications?
8. Could a biosimilar product for this case study be developed fully *in vitro*?

## Workshop case studies

### Case study 1: Anti-ADAMTS-5 mAb

#### Background

ADAMTS-5 is a member of the ADAMTS (a disintegrin and metalloproteinase with thrombospondin motifs) protein family. It is an aggrecanase that degrades the aggrecan component of articular cartilage, making it an attractive target for osteoarthritis. Anti-ADAMTS-5 is a humanized, IgG1 Fc-disabled mAb that selectively inhibits ADAMTS-5 activity in the mouse, rat and NHP, including the cynomolgus monkey. The expression pattern of ADAMTS-5 shows that it is expressed in articular cartilage and surrounding joint tissues, but also many other tissues including arterial smooth muscle cells, mesothelium lining the peritoneal, pericardial and pleural cavities, smooth muscle cells in bronchi and pancreatic ducts, glomerular mesangial cells in the kidney, dorsal root ganglia, and Schwann cells.^32^ ADAMTS-5 knockout mice show enlarged cardiac valves associated with accumulation of versican, which persists in adult mice.^33^

#### Non-clinical development program and toxicology findings

Non-clinical toxicology studies were conducted in the cynomolgus monkey and also the Wistar Han rat (see Table 2). Mechanistic investigational studies were performed *in vitro* (see Table 3).

**Table 2.** *In vivo* studies for anti-ADAMTS-5.

Species	Study^1^	Dose/s	Group sex and size	Findings
Cynomolgus monkey	4 week DRF	0 mg/kg (iv and sc)	3F/group	Minimal focal endocardial hemorrhage in the left ventricle in 2 of 3 monkeys in 300 mg/kg/dose
		30 mg/kg (sc)300 mg/kg (iv)		Uncertain relationship to test article due to high background incidence
Cynomolgus monkey	8 week GLP toxicology	0 mg/kg (iv and sc)	3M+3F/group	No cardiac findings (including ECG and cardiac troponin)
		30 mg/kg (sc)		Abnormal fecal consistency and decreased inorganic phosphorus concentrations noted in monkeys given 300 mg/kg/dose
		100mg/kg (iv)		
		300 mg/kg (iv)		
Wistar Han rat	4 week DRF	0 mg/kg (iv and sc)	4M/group 3M/group for TK	No noteworthy findings
		10 mg/kg (iv)		
		10 mg/kg (sc)		
		300 mg/kg (iv)		
Wistar Han rat	8 week GLP toxicology	0 mg/kg (iv and sc)	10M+10F/group 3M+3F/group for TK	Decreased mean body weight gain and food consumption in males given ≥100 mg/kg/week iv.
		30 mg/kg (sc)		
		100mg/kg (iv)		
		300 mg/kg (iv)		
Cynomolgus monkey	Single Dose GLP Cardiovascular/Respiratory	0 mg/kg (iv and sc)	3F/group	Dose dependent ST segment elevation at 30 and 300 mg/kg on Days 1, 7, 14 and 21
	Jacketed ECG and telemetry device for arterial blood pressure	30 mg/kg (sc) 300 mg/kg (iv)		Other ECG waveform abnormalities
				– 30 mg/kg – intraventricular conduction delay and increased frequency of isolated premature ventricular contractions (PVCs, including bigeminy and couplets), occurring mainly on Day 1
				– 300 mg/kg - increased frequency of isolated PVCs and 1 occurrence of R on T PVC on Days 1 and 7
				↑ in mean arterial pressure (MAP)
				– 30 mg/kg - increase on Day 21 with max. increase of ∼31 mmHg or 30% vs vehicle
				– 300 mg/kg - increase on Days 14 and 21 with max. increases of ∼23 mmHg or 22% vs vehicle. This dose also produced increased Cardiac Work with increases of 39–48%
				At ∼7 months post-dose (non-GLP), ST segment elevations still present
				– In heart tissue, there were no test article-related microscopic findings or differences in IHC for Connexin 43 (gap junctions), versican proteolytic fragments (ECM) or GSK2394002
				– MAP was not evaluated because telemetry implants were no longer operational
				No consistent effects on HR or PR, QRS or QTc interval durations
				No effects on ventilatory parameters
			
			
			
			
			
Wistar Han rat	Investigative Repeat Dose Cardiovascular	0 mg/kg (iv) 300 mg/kg (iv)	4M/group	Single dose at 300 mg/kg in conscious rat did not produce any waveform abnormalities or arrhythmias (including ST segment changes) or arterial pressure changes on Days 1, 8 or 15 (continuous 24 hr monitoring)
	Telemetry device for arterial pressure, heart rate, ECG (intervals and waveform abnormalities/arrhythmias), and body temperature			
Cynomolgus monkey	Repeat Dose GLP Cardiovascular study to identify NOEL	0 mg/kg (iv)	3M+3F/group	0.3 mg/kg
		0.3 mg/kg (iv)		– Increased MAP following the 3rd dose (up to 8 mmHg)
		3 mg/kg (iv)		– One monkey had multiple episodes (31) of non-sustained ventricular tachycardia (NSVT) following the 1st dose
				Three mg/kg
				– Increased MAP following 2nd dose (up to 9 mmHg or 9% of vehicle) and throughout the remainder of the study (Day 70)
				– Decreased HR between 4–16 hrs following 1st and 2nd dose (up to 19 bpm or ∼13%)
				No evidence of ST segment elevation at 0.3 or 3 mg/kg
Cynomolgus Monkey	Vehicle Investigative Study	Veh (iv) or Veh (iv) with 10X Tween™ 80 (0.2% w/v) or 10X L-arginine (10% w/v)	4M+4F (cross over)	No arrhythmic effects with vehicle at 10X Tween™ 80 (0.2% w/v) or 10X L-arginine (10% w/v)

All studies were conducted in accordance with the GSK Policy on the Care, Welfare and Treatment of Laboratory Animals and were reviewed the Institutional Animal Care and Use Committee either at GSK or by the ethical review process at the institution where the work was performed.

**Table 3.** *In vitro *studies for anti-ADAMTS-5.

Species	Study	Findings
Rabbit	Rabbit cardiac wedge assay	No arrhythmogenic activity or ST segment elevation observed at concentrations up to 500 µg/mL
Human	Human CV ion channel assays (hERG, NaV1.5 and CaV1.2)	No difference from vehicle at concentrations up to 500 µg/mL (note – acetate inhibited NaV1.5)
	Selectivity profiling	Fully selective for ADAMTS-5 within class (ADAMTS family and MMPs)
		ProtoArray™ screen (>9400 proteins) – Only specific binding to ADAMTS5 observed
		Tissue cross reactivity – no signal, anti-ADAMTS-5 not a good immunohistochemistry reagent

**Figure 2.**
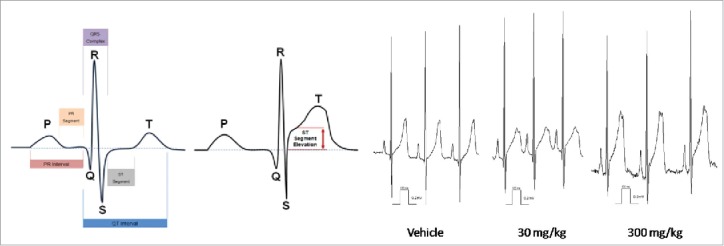
Dose-dependent ST segment elevation with anti-ADAMTS-5 in cynomolgus monkey.

Cardiovascular effects (mean arterial pressure increase and ECG waveform abnormalities) were observed in monkey with anti-ADAMTS-5 in doses above 0.3 mg/kg. ST segment elevations were still detected 7 months post-dose (see Fig. 2). Pre-existing knowledge of the target (i.e., phenotypes of knockout mice) suggested that there was a potential cardiovascular developmental risk, but did not predict the adverse electrophysiology. Furthermore, *in vitro* assays, such as the rabbit cardiac wedge assay and human CV ion channel assays did not detect the risk. There were no cardiovascular changes detected in the rat.

#### Workshop breakout discussion

Attendees at the workshop were asked to consider the questions in Table 1. The consensus of the breakout group was that the cardiovascular effects observed in the nonclinical studies were not predictable based on the mechanism of action of anti-ADAMTS-5. Due to the target expression in the cardiovascular tissue, the heart was identified as a potential target organ; however, the observed effects (hemorrhage in the initial non-GLP study, acute arrhythmias, persistent ST segment elevations and dose-dependent delayed onset increase in blood pressure) could not have been predicted. The only adverse effect that could possibly be attributed to a background lesion in the animal was the hemorrhage.

Because a main focus of the workshop was the increased use of emerging technologies, the ability of *in vitro* approaches to identify the adverse effects was discussed. For anti-ADAMTS-5, the currently available *in vitro* tools were considered unable to predict the observed effects, as there are no *in vitro* models for hemodynamics or potential secondary pharmacologic effects, such as those on the extracellular matrix, that were relevant to this case study.

Evaluating the totality of the *in vivo* findings for this case study highlighted that the most useful information was obtained from the dedicated safety pharmacology evaluation in cynomolgus monkeys. Limitations of cardiovascular measurements conducted as part of the repeat-dose GLP toxicology study were also discussed and considered insufficient to detect the ECG findings observed in the safety pharmacology study. This was thought to be due to readings being taken at one minute intervals rather than over 24 hours as in the dedicated safety pharmacology study, and that there was no measurement of blood pressure included.

### Case study 2: Anti-DLL4 mAb

#### Background

Delta-like ligand 4 (DLL4) is a ligand in the Notch family of endothelial cell receptors that functions to control the balance of tip and stalk cells during normal vascular development. Heterozygous DLL4 knockout mice show embryonic lethality due to vascular abnormalities, and more recent experiments show that conditional knockout of Notch 1 can also lead to the development of vascular tumors in mice.^34^^,^^35^ Targeting DLL4 with an antibody (anti-DLL4 IgG1) yielded robust anti-tumor activity in several nonclinical models, making DLL4 an attractive therapeutic target.^36-39^ The findings from *in vivo* toxicology studies using an anti-DLL4 IgG1 mAb, however, raised serious safety concerns that were considered target-related based on the known expression and function of DLL4, although the specific manifestations of toxicity observed following repeated treatment had not been predicted *a priori* from pre-existing information alone. Given the potential promise of DLL4 inhibition for anti-tumor activity, a second approach was then undertaken by engineering a Fab’2 fragment to target DLL4 (rather than inhibition of DLL4 with the full IgG1 antibody). Although the Fab’2 approach ultimately mitigated some of the target-related toxicities seen with the IgG1 molecule, other unexpected target-related toxicities were revealed and resulted in termination of the program before FIH studies.^40^

#### Non-clinical development program and toxicology findings

Various *in vitro* studies were performed with anti-DLL4 IgG1 and Fab’2 candidate molecules to confirm effective DLL4 pathway inhibition, including a mouse retinal explant model and a human umbilical vein endothelial cell potency assay to demonstrate the expected pharmacology of DLL4 inhibition of nonproductive angiogenesis and endothelial cell proliferation, respectively. Biacore data confirmed similar potency of anti-DLL4 binding across mice, rats, NHP and humans. GLP toxicology studies were therefore conducted using the Sprague Dawley rat and the cynomolgus monkey for both the IgG1 mAb and the Fab’2 candidate molecules, to evaluate toxicity in 2 species (rodent and non-rodent) as per ICH S6.^41^ Findings observed following anti-DLL4 IgG1 mAb administration in both species included marked atrophy of centrilobular hepatic cords, sinusoidal dilation, bile ductular proliferation, elevated liver function tests and decreased red blood cells. However, the severity and incidence of these findings differed between species, such that the liver findings were more severe in the rat relative to the cynomolgus monkey, and the decrease in red blood cells was more severe in the cynomolgus monkey. There were also additional findings in the rat that were not observed in NHPs, including proliferative vascular neoplasms in the skin, lung, and heart (Table 4).

**Table 4.** *In vivo* studies for anti-DLL4 IgG1 mAb.

Species	Study	Dose/s	Group sex and size	Findings
Sprague Dawley rat	8 week GLP toxicology	0 mg/kg (iv)	10M+10F/group and 5M+5F recovery/group and 6M+6F TK satellites/group	Liver findings: Hepatic centrilobular to bridging sinusoidal dilation
		1 mg/kg (iv)		Decreased red blood cells (monitorable)
		3 mg/kg (iv)		Proliferative vascular neoplasms
		10 mg/kg (iv)		– Skin, lung, and heart lesions seen only in rats
		30 mg/kg (iv)		
Cynomolgus monkey	8 week GLP toxicology with 12 week recovery	0 mg/kg (iv)	3M+3F/group and 2M+2F recovery/group and 2M+2F for telemetry on the control and high dose group	Liver findings: Hepatic centrilobular to bridging sinusoidal dilation
		0.2 mg/kg (iv)		Decreased red blood cells (monitorable)
		0.8 mg/kg (iv)		
		3 mg/kg (iv)		
		12 mg/kg (iv)		

To further evaluate whether the DLL4 pathway could be therapeutically targeted without compromising safety, a Fab’2 fragment of the anti-DLL4 antibody with more rapid clearance and shorter half-life than the original IgG1 antibody was developed.^34^ GLP toxicology studies with the new molecule showed an improved toxicity profile regarding the liver, red blood cell loss, and occurrence of proliferative vascular lesions. However, new findings were also identified with the Fab’2 fragment in the heart and lung of both species that were suggestive of pulmonary hypertension and considered related to DLL4 inhibition (Table 5). Together, these findings suggest that inhibiting the DLL4 pathway under different conditions (e.g., different exposure regimens) may lead to differential and unexpected findings *in vivo.*

**Table 5.** *In vivo *studies for anti-DLL4 Fab’2 fragment.

Species	Study	Dose/s	Group sex and size	Findings
Sprague Dawley rat	8 week GLP toxicology	0 mg/kg (iv)	10M+10F/group and 5M+5F recovery/group and 6M+6F TK satellites/group	Liver findings: Decreased severity compared with IgG, minimal changes observed at low doses (up to 10 mg/kg)
		3 mg/kg (iv)		Acceptable hematology profile
		10 mg/kg (iv)		Proliferative vascular neoplasms not observed
		100 mg/kg (iv)		New findings: Vascular proliferative/degenerative changes in the heart and lung potentially related to pulmonary hypertension
		30 mg/kg (iv)		
Cynomolgus monkey	8 week GLP toxicology with 12 week recovery	0 mg/kg (iv)	3M+3F/group and 2M+2F recovery/group and 2M+2F for telemetry on the control and high dose group	Liver findings: Decreased severity compared with IgG
		5 mg/kg (iv)		Acceptable hematology profile
		15 mg/kg (iv)		New findings: Vascular proliferative/degenerative changes in the heart and lung potentially related to pulmonary hypertension
		50 mg/kg (iv)		

#### Workshop breakout discussion

At the start of the anti-DLL4 development program, the hypothesis was that the DLL4 pathway was only active in tumor, rather than normal vasculature, and that the effects would be similar to the approved angiogenesis inhibitor, bevacizumab (Avastin®). Attendees were asked to consider the questions in Table 1. The breakout group considered that clinically relevant findings in general repeat dose toxicity studies were related to the mechanism of action of the mAb and the majority of the participants agreed with this. However, it was agreed that only some of the findings, for example those that were characteristic of bevacizumab, were predictable *a priori*. There were significant, clinically relevant toxicities (e.g., liver sinusoidal changes, heart and lung changes and anemia) that the participants thought were not predictable before *in vivo* studies. Importantly, the dose-related toxicities seen in rats and monkeys resulted in a decision to terminate both anti-DLL4 molecules before FIH studies.

Only a third (30%) of the participants thought that some of these clinically relevant findings could be predictable in the future using existing/future *in vitro* technologies and systems biology approaches. Important gaps identified in currently available *in vitro* approaches included difficulty in modeling paracrine effects between at least 2 inter-regulated cell types, as well as in modeling potential hemodynamic effects that could lead to pathway-related changes only apparent in an *in vivo* setting. For example, data from human cardiomyocytes or hepatocytes would be limited as anti-DLL4 may be acting at the endothelium and the sinusoids, respectively. A primary limitation of current *in vitro* systems is therefore associated with the ability to integrate multiple cell types (e.g., stalk cells, tip cells and epithelia), with appropriate tissue architecture and hemodynamics, to tackle questions relating to potential *in vivo* physiologic effects of the drug. The potential for Notch signaling studies to provide information on cross-species potency and add value to the overall program was also discussed. Although signaling studies were considered to be of limited value in predicting *in vivo* toxicity in this case, the group were interested in evaluating the potential for 3-dimensional (3D) tissue models, including novel microfluidic and dynamic flow systems currently in development (e.g., as described in ref. 42), to predict toxicity within a more physiologic organ architecture. Given the liver phenotype observed following administration of anti-DLL4 *in vivo*, these more complex organ models were thought to hold potentially greater promise for accurate prediction of toxicities that may only be reproduced in the context of relevant sinusoidal architecture or hemodynamic changes on vascular endothelial cells. The breakout group agreed that if development of an anti-DLL4 molecule had continued further, a chronic toxicity study in the rat alone (rather than the cynomolgus monkey or in both species) may be useful to further identify potential effects with long-term treatment, as the rat and monkey exhibited similar toxicity profiles, and it would therefore be appropriate to conduct additional nonclinical studies in the lower-order species to limit NHP use.^41^

### Case study 3: Anti-amyloid β mAb

#### Background

The anti-amyloid β mAb discussed in case study 3 is a humanized monoclonal IgG1 antibody. It is specific for a conformation of amyloid β (Aβ) protein oligomer and binds the oligomer with high selectivity compared with other Aβ conformations such as fibrils or monomer. This high selectivity for the Aβ oligomer was predicted to result in improved efficacy and reduced side effects in the treatment of Alzheimer's Disease. The target antigen is primarily present in Alzheimer's Disease state, and is essentially undetectable in normal animals. Unexpected cross-reactivity of the anti-amyloid β mAb to a plasma protein cytokine resulted in preclinical toxicity in NHPs.^43^ Studies by Vugmeyster et al., which used a humanized anti-amyloid antibody against amino acids 3-6 of primate amyloid beta, and which was published after this work had been performed, demonstrated off-target binding to fibrinogen which was shown to slow clearance.^44^

#### Non-clinical development program and toxicology findings

The *in vivo* and *in vitro* studies are summarized in Tables 6 and 7, respectively. The anti-amyloid β mAb was found to normalize synaptic function and improve cognitive function in amyloid precursor protein (APP) transgenic mouse model of Alzheimer's Disease. No side effects were observed in a 4-week non-clinical APP mouse study. A tissue cross-reactivity panel in monkeys and humans showed no noticeable binding, and there was no binding to human peripheral blood cells. No side effects were observed following a single low-dose administration in a cynomolgus monkey pharmacokinetics study, but severe toxicological effects were observed in a 13-week repeat-dose cynomolgus monkey study. At low doses (20 and 60 mg/kg/week), thrombocytopenia and vasculature changes (medial hypertrophy and thrombosis) were observed, along with neuron loss and microhemorrhages in the brain. Higher doses (120 and 200 mg/kg) caused an acute infusion reaction upon the first dose, with lethal consequences. The rapid onset after the first dose at 200 mg/kg was indicative of an effect initiated by binding of anti-amyloid β mAb to an already present plasma antigen. Further evaluations identified unintended off-target binding to a specific plasma protein cytokine that is released from activated platelets and has strong chemoattractant properties for neutrophils and fibroblasts (Table 7). The binding of the anti-amyloid β mAb to the plasma protein resembles the pathological function of Heparin-induced Thrombocytopenia (HIT), leading to HIT-like symptoms such as thrombocytopenia. Thus, the off-target binding was consistent with the thrombocytopenia, vascular changes and infusion reactions that were observed in the cynomolgus monkey toxicity study.

**Table 6.** *In vivo *studies for anti-amyloid β mAb.

Species	Study	Dose/s	Group sex and size	Findings
APP-transgenic mouse model	Histopathological evaluation of select tissues in a pharmacology study.	0.5 mg/mouse/ week (ip)	15M/group; 7-11 animals/group available for histopathological evaluation	Histological changes (neoplasia, renal changes, degenerative joint disease, skeletal muscle de/re-generation) were considered due to aging or potential background changes in mouse strain and not due to antibody treatment.
APP-transgenic mouse model	4 week GLP toxicology	0 mg/kg (iv and sc)	12M+12F/group and 6M+6F recovery/group and 18M+18F TK satellites/group. Plus additional TK groups for control and 200 mg/kg (iv) with 12 weeks treatment.	Up to 200 mg/kg for 4 weeks was well tolerated. 200 mg/kg for 12 weeks was well tolerated in animals for TK analysis. Anti-drug antibodies were detected in 62% of antibody-dosed animals.
		60 mg/kg (iv)		NOAEL = 200 mg/kg.
		200 mg/kg (sc)		
		200 mg/kg (iv)		
Cynomolgus monkey	PK study	5 mg/kg single dose (iv and sc)		t ½ ∼8 d. Low clearance and volume of distribution. No adverse or notable effects observed.
Cynomolgus monkey	13 week GLP toxicology	0 mg/kg (iv and sc)	5M+5F	Low doses (20 and 60 mg/kg): Thrombocytopenia (decreased platelets), pulmonary vasculature changes (medial hypertrophy, thrombosis), lung findings (multifocal hemorrhages, interstitial fibrosis).
		20 mg/kg (iv)	120 mg/kg (iv) 1M	High doses (120 and 200 mg/kg): Acute infusion reaction (5-10 min post-dose) with lethal consequence at 200 mg/kg.
		60 mg/kg (iv and sc)	200 mg/kg (iv) 4M	
		120 mg/kg (iv)		
		200 mg/kg (iv)		
Cynomolgus monkey	Exploratory toxicity study	0 mg/kg (iv bolus)	1M+1F	IV infusion (1 hour) produced a less severe acute infusion reaction compared with iv bolus administration at the same dose level. Acute infusion reaction ≥ 60 mg/kg included complement activation. No effects on cytokines, coagulation factors or ECG's. Histopathologic effects observable after 4 weeks of dosing (thrombotic and/or arterial changes in brain, lung and injection sites).
		2 mg/kg (iv bolus)		NOAEL = 2 mg/kg
		60 mg/kg (iv bolus)		
		120 mg/kg (iv infusion or bolus)		
		200 mg/kg (iv infusion)

**Table 7.** *In vitro *studies for anti-amyloid β mAb.

Species	Study	Findings
Human	Cytokine release	Cytokine release assay in whole human blood (IL-1ra, IL-1b, IL-6, hTNFa, IL-8) was positive for IL-8 release at ≥ 10 µg/mL.
Human	PBMC binding	No PBMC staining at 10 or 30 µg/mL.
Human, cynomolgus monkey, Tg mice (3 donors each)	Tissue cross-reactivity with FITC-labeled Ab (GLP)	No noticeable binding.
Human and cynomolgus monkey	Platelet binding (FACS)	No binding to platelets in human blood (Fc mutation and wt alike). No binding to CD41+ platelets in cynomolgus blood (Fc mutation and wt alike). Some binding to CD41- “platelet-like” cells in cynomolgus blood (Fc mutation and wt alike).
Human, cynomolgus monkey, mouse and rat	Serum binding (ELISA)	Concentration-dependent binding to serum component of species tested (Fc mutation and wt alike). No binding of murine Ab to any species. Binding to serum component is not CDR/target mediated; suggests that binding is not Fc mediated.

#### Workshop breakout discussion

The consensus of the breakout group was that the effects observed in the nonclinical studies, particularly the thrombocytopenia and vascular effects, were not predictable based on the mechanism of action of anti-amyloid β mAb. The off-target binding could not have been identified via any other means of *in vitro* testing available at the time (e.g., cytokine release, whole blood binding assays), though it may now be possible to identify potential off-target binding through an extended *in vitro* binding cascade. A wider range of *in vitro* approaches may have aided the prediction of the effects observed, for example to screen for plasma or serum component binding before *in vivo* studies.

Over half of the participants (53%) agreed that in future the observed effects could be predictable with greater use of new/existing *in vitro* technologies, whereas a third of the participants (30%) disagreed, with the remaining participants undecided. Since the off-target cross-reactivity was only present in human and cynomolgus plasma, not in mouse, rat or dog plasma, this case demonstrates the importance of testing the safety of therapeutic antibodies in a species relevant for both on-target and off-target binding.

## Workshop consensus

A voting system was used throughout the workshop to gauge participant opinion, and the results are presented and discussed below.

### Opportunities for in vitro technologies

The presentations and discussion at the workshop inspired many participants to think differently about how new technologies could be integrated into drug development approaches for biotherapeutics, and most (83%) thought that industry should use more *in vitro* approaches wherever feasible to reduce animal use. Almost all participants (95%) agreed that there were specific situations where *in vitro* data from human systems was more important than *in vivo* data from animal studies (e.g., cytokine release (TGN1412)). When asked whether regulators would accept *in vitro* data in lieu of some *in vivo* data to support FIH dosing, 55% of participants were sceptical, pointing toward the need for the scientific community to generate convincing data on the validity of *in vitro* models for human risk assessment. The most likely aspect of nonclinical toxicology for biopharmaceuticals to be replaced by *in vitro* approaches was thought to be carcinogenicity (50%), followed by general toxicology (29%) then reproductive toxicology (11%) and juvenile toxicology (11%). The need to improve screening for off-target tissue binding before *in vivo* studies (and to potentially replace GLP tissue cross-reactivity studies in the future) was also identified as an area ripe for improvement (83% agreement).

### Value of the in vivo studies

The majority of participants (86%) agreed that while some *in vivo* nonclinical findings resulting in termination of projects may have been false positives and not relevant to humans, these were difficult to predict and it was unlikely that such risk would readily be taken to enable these drugs to enter the clinic. Many companies also reported increased requests for juvenile toxicity studies to support pediatric clinical development. Participants generally agreed that these studies were rarely or never needed to support pediatric indications for age 6-12 y (never (61%), sometimes (34%) and always (5%)). There was a slight shift in participant experience for the 2-6 y age range, with half workshop participants voting that these studies were rarely or never needed (never (49%), sometimes (46%) and always (5%)). Better integration of information from general toxicology studies, clinical data from adult patients, modeling and systems biology approaches (such as those currently used for dose calculation) was considered likely to supersede the need for juvenile toxicology studies (73% agreed). This position is supported in the newly developed ICH S11 ‘Non-clinical Safety Testing in Support of Development of Pediatric Medicines’ concept paper that provides guidance and direction on the nonclinical safety studies needed to support a pediatric development program.^45^ Companies had also experienced regulatory requests to assess bone quality endpoints in ovariectomized NHPs for certain classes of drugs, although participants generally agreed that this type of study did not add value for human risk assessment (54% agreed, 17% disagreed, 29% don't know).

One approach to refine *in vivo* nonclinical development programmes in the future may be to conduct a single toxicology study to enable clinical trials. This study would not need to be longer than 6 months as long-term chronic toxicology studies (9/12 months) do not often detect additional/new toxicities compared with the shorter-term studies, as agreed upon with the ICH S6 Addendum.^11^^,^^41^^,^^46^ More data are required to assess whether there are more scientifically justified opportunities to conduct a single study of 12 weeks for FIH clinical trials other than those for serious life-threatening conditions. Indeed, in cases where the mAb is directed against a target that is minimally expressed in naïve animals or does not play a role in normally physiology, a short-term study of 1 month duration may be sufficient. The current ICH guidelines and regulatory environment should be amenable to this, as the guidelines are meant to act as a *guide*, and do not currently dictate study duration, aside from that they should be based on the intended duration of clinical exposure and disease indication. Furthermore, regulators will allow deviation from the guidelines, taking scientific rationale into account on a case-by-case basis.

### Data-sharing and transparency

There was consensus from regulators and industry that the workshop had provided a useful forum for open discussion of case studies that were not in the public domain, and participants agreed that there was value in increasing availability of data from terminated biotherapeutics to regulators (91% agreed). There would also be value in making this information available to other industry stakeholders to reduce redundancy in animal studies and potentially enable broader innovation across the industry. However, the significant challenge identified would be achieving this in practice, as only 55% of attendees had confidence that they could persuade their companies to see the value of releasing such cases into the public domain due to competitive and intellectual property concerns. Furthermore, there are often difficulties in publishing this sort of data if the project has been terminated before establishing the exact cause of the toxicity.

The ongoing challenge for regulators is that they only see the few molecules that companies choose to advance into clinical trials, which are typically much less likely to have associated or severe toxicities, as the most concerning candidate molecules/targets have often been terminated before any regulatory interactions. In developing a future vision for mAb development, one important aspect for consideration is a continued evolution of regulatory practice and policy. For example, the amount of knowledge and data that is generated as a by-product of the regulatory submissions process is critical to ensure future strategy is directed and informed by science through a broad evidence-base. Although information collected in surveys is useful for certain purposes, such as in developing recommendations on good practices, in this case the importance of detailed specific case study information was acknowledged.

## Discussion and future work

The scientific and regulatory community clearly share a vision for continued evaluation and integration of emerging technologies to reduce and refine animal use for biotherapeutic mAb development. However, there are still several barriers that must be recognized and overcome to make this a reality. When individual case studies were discussed and retrospectively analyzed, the ability of existing *in vitro* and *in silico* technologies to detect or predict toxicities observed in *in vivo* studies was noted as lacking (summary in Table 8). Some of the observed *in vivo* effects, such as changes in blood pressure or paracrine effects, would not have been predicted using currently available technologies; therefore, the challenge for the future will be to advance and apply novel technologies that have the capability to more closely represent the *in vivo* situation.

**Table 8.** Summary of case-study data and the ability of existing *in vitro* and *in silico* technologies to detect or predict toxicities observed in the *in vivo* studies.

	Anti-ADAMTS-5 mAb	Anti-DLL4 mAb	Anti-amyloid β mAb
Were findings based on the mechanism of action of the mAb?	Yes	Yes	No
Were findings predictable?	Although cardiovascular binding was detected the toxicological effects were not predictable	Some findings were predictable but additional clinically relevant findings were detected that were not predictable	No
Is an *in vitro* model available now or in the near future?	Not for hemodynamics or secondary effects (e.g., extracellular matrix interactions)	Generally no, due to the need to model paracrine effects between multiple cell types	After off-target binding was identified, *in vitro* screening could be used to select a better molecule without off-target binding, but still need some *in vivo* studies to test that the lack of off-target binding does not cause *in vivo* effects.
What was the impact of the *in vivo* studies?	Clinically relevant toxicity was only identified in the stand alone safety pharmacology study	Data from *in vivo* studies were used to make a decision to terminate the mAb and Fab’2 programs.	Data from the NHP study identified cross-reactivity that would not have been identified in mouse, rat or dog. Clinical studies in humans were not conducted with this antibody.

The majority of clinically relevant findings for mAbs are based on their mechanism of action. However, the toxicities presented in the case studies were, in general, not predictable before *in vivo* studies despite their relationship to the pharmacological action of the mAb. It is also important to note that many associated clinical toxicities such as some cancers, progressive multifocal leukoencephalopathy and infection, are so rare that they are not realistically detected in any *in vitro* or *in vivo* study. Currently, the field remains insufficiently confident in the ability of *in vitro* models to capture unpredictable toxicological findings as highlighted in the case studies, although there is much enthusiasm, commitment, and perceived potential for the industry to work towards this aim. Significant activity will be required to progress this field to be able to confidently predict unexpected toxicities from *in vitro* models. The development of more sophisticated and relevant *in vitro* technologies for safety assessment of mAbs may need to be more case-dependent, to take in to account their innate complexity, diversity and size, as well as their specific mechanism of action. A major recommendation of the participants at the workshop was for the establishment of a framework that could improve pre-competitive data-sharing between companies developing biologic products. Increased communication and data-sharing would enhance progress, increase understanding between industry and regulators, and support advancement toward common goals. The challenge faced in developing such a framework is in providing incentives for companies to share data on terminated compounds, which could take the form of individual company publications, cross-company initiatives, consideration of coordination with the EMA safe harbour effort, as well as development of an online journal, database or repository that would provide an easily accessible platform to share additional case studies. The NC3Rs could potentially serve as an honest broker to take this type of initiative forward; Biosafe (a committee within the Biotechnology Innovation Organization, a trade association for biotechnology-related organizations globally) is also working to collect several similar case studies that can be published and presented to the FDA.

As well as consideration of the potential for emerging technologies, the value of the existing *in vivo* studies was also discussed at the workshop. A topic identified with the potential to unnecessarily increase NHP use in the future was an increase in juvenile toxicity studies as default practice to support mAb development in pediatric populations, due to regulatory perception within companies and previous requests from the Pediatric Committee (PDCO/EMA). The regulatory requests were not always deemed to be scientifically driven and many participants disagreed that juvenile toxicity studies were necessary to inform pediatric safety in many cases, as there is potential to better utilize and integrate information from general toxicology studies and clinical data from adult patients. Since the discussions at the workshop, the guidance published in the ICH S11 concept paper may alleviate some of these concerns.^45^ However, to prevent unnecessary conduct of these studies as the general rule, a data-sharing initiative will be needed to evaluate whether juvenile toxicity studies in animals provide any additional clinically relevant information, and if so, in what circumstances.

A future vision for mAb development is one in which fewer animals are used, but where the data obtained are more predictive of human safety. Therefore the typical approach to safety assessment of mAbs was considered. Typically two studies, one to support FIH clinical studies (IND-enabling) and one to support registration, are performed during mAb development. In some cases for oncology indications, a single study may suffice.^47^ However, an alternate approach could be to use a single, comprehensive *in vivo* study for the majority of mAbs that includes, in addition to toxicity endpoints, relevant pharmacodynamic, biomarker and potentially safety pharmacology endpoints. It has been argued that new clinically relevant findings are rarely identified in long-term studies that were not observed or could not have been predicted from the short-term study.^11^^,^^46^ Future work will involve whether both the 12-week and 26-week studies are of value in detecting clinically relevant findings. Of course, it is often the rare cases that ultimately drive regulations to ensure adequate safety in clinical trials. One recommendation for further progress in this area is to generate an evidence-base to help determine the frequency and types of toxicity that are observed only in long-term studies (6 months) compared with the shorter-term studies (1-3 months), and to explore whether these risks might be predicted in advance for specific types of targets. For example, the majority of participants felt that products such as cytokines and other soluble factors could be safely approved based on the IND-enabling toxicology study with no chronic toxicology (54% agreed, 32% disagreed, 15% don't know).

## Conclusions

A number of areas have been identified for future resource and investment that are critical to reach a scientifically driven vision for future biotherapeutic mAb development. These include the development and increased use of emerging technologies such as cell and tissue-based technologies that are suitable for mAb development, and opportunities to waive chronic and juvenile toxicity studies. Currently, many of the emerging technologies are being developed with small molecule new molecular entities in mind, rather than biologics, and a shift toward application for biologics is needed for these technologies to play a role in mAb development (e.g., development of hemodynamic models or paracrine effects models is lacking). A number of organizations have an interest in progressing this area across the regulatory, industry and public sectors and the time is right for collaboration to shape future investment, data-sharing activities and technology development in this area. The aim of our workshop was to contemplate what will be possible in the next 10 y rather than focus on our current capabilities. It is clear that there are opportunities to improve mAb development, but this will not happen without the collective knowledge, experience and dedication of experienced drug safety professionals and a more open-minded approach to mAb development.
